# A meta-review of literature reviews assessing the capacity of patients with severe mental disorders to make decisions about their healthcare

**DOI:** 10.1186/s12888-020-02756-0

**Published:** 2020-06-30

**Authors:** A. Calcedo-Barba, A. Fructuoso, J. Martinez-Raga, S. Paz, M. Sánchez de Carmona, E. Vicens

**Affiliations:** 1 Department of Psychiatry, Hospital Gregorio Marañón; Medical School, Universidad Complutense de Madrid, Doctor Esquerdo 46, 28007 Madrid, Spain; 2 Adult Psychiatry Service and Geneva Penal Medicine Division, Geneva University Hospitals, Puplinge, Switzerland; 3 Psychiatry Service, University Hospital Doctor Peset, University of Valencia, Valencia, Spain; 4 SmartWriting4U, Valencia, Spain; 5 Medical School, Universidad Anáhuac, Mexico City, Mexico; 6 Department of Psychiatry, Parc Sanitari Sant Joan de Déu, Barcelona, Spain

**Keywords:** Decision making capacity, Mental disorder, Schizophrenia, Bipolar disorder, Literature review, Meta-review

## Abstract

**Background:**

Determining the mental capacity of psychiatric patients for making healthcare related decisions is crucial in clinical practice. This meta-review of review articles comprehensively examines the current evidence on the capacity of patients with a mental illness to make medical care decisions.

**Methods:**

Systematic review of review articles following PRISMA recommendations. PubMed, Scopus, CINAHL and PsycInfo were electronically searched up to 31 January 2020. Free text searches and medical subject headings were combined to identify literature reviews and meta-analyses published in English, and summarising studies on the capacity of patients with serious mental illnesses to make healthcare and treatment related decisions, conducted in any clinical setting and with a quantitative synthesis of results. Publications were selected as per inclusion and exclusion criteria. The AMSTAR II tool was used to assess the quality of reviews.

**Results:**

Eleven publications were reviewed. Variability on methods across studies makes it difficult to precisely estimate the prevalence of decision-making capacity in patients with mental disorders. Nonetheless, up to three-quarters of psychiatric patients, including individuals with serious illnesses such as schizophrenia or bipolar disorder may have capacity to make medical decisions in the context of their illness. Most evidence comes from studies conducted in the hospital setting; much less information exists on the healthcare decision making capacity of mental disorder patients while in the community. Stable psychiatric and non-psychiatric patients may have a similar capacity to make healthcare related decisions. Patients with a mental illness have capacity to judge risk-reward situations and to adequately decide about the important treatment outcomes. Different symptoms may impair different domains of the decisional capacity of psychotic patients. Decisional capacity impairments in psychotic patients are temporal, identifiable, and responsive to interventions directed towards simplifying information, encouraging training and shared decision making. The publications complied satisfactorily with the AMSTAR II critical domains.

**Conclusions:**

Whilst impairments in decision-making capacity may exist, most patients with a severe mental disorder, such as schizophrenia or bipolar disorder are able to make rational decisions about their healthcare. Best practice strategies should incorporate interventions to help mentally ill patients grow into the voluntary and safe use of medications.

## Background

In 1995, Appelbaum and Grisso stated that competence to consent to treatment relied on four legal standards: the ability to communicate a choice; the ability to understand relevant information; the ability to appreciate the situation and its likely consequences; and the ability to manipulate information rationally [1]. In healthcare, the capacity to make decisions regarding treatment is closely related to the autonomy, the exercise of self-governance, and the ability of an individual to take intentional actions [2]. The capacity to consent to treatment is often used in the clinical assessment of the ability to engage in authentic autonomous decision-making, a fundamental element of a person’s dignity and rights [3].

Assessment of mental capacity has become a key component of daily clinical practice [4, 5]. Mental health legislation and medical ethics increasingly require physicians to empower patients to make decisions, and to respect the patient’s wishes with regard to accepting or refusing therapy [4, 6]. However, it has been reported that coercive treatment, involuntary hospitalisations and medications are currently overused [7]; this has a direct negative impact on patients’ adherence to treatment and on their engagement and participation in shared decision-making with their healthcare professionals [8].

An increasing number of publications are assessing decision-making capacity in mental health. However, comparisons and contrasts of the findings of these articles are lacking and it becomes difficult to draw clear conclusions on what is the actual capacity of individuals with serious mental illnesses to make decisions about their healthcare and treatments [9]. This meta-review of review articles was designed as a comprehensive synthesis of the current state of knowledge in the field, with the aim of assessing the available evidence on the decision-making capacity of patients with various mental illnesses (especially schizophrenia, psychosis and bipolar disorder) with regard to the management of their disease and their treatment. The review compares the conclusions of various comprehensive publications, discusses the strength of these conclusions, and identifies existing gaps in the evidence.

## Methods

The review of the literature was conducted in accordance with the Preferred Reporting Items for Systematic Review and Meta-Analysis (PRISMA) guidelines [10]. A series of steps, including the definition of the search strategy, identification and selection of publications, data extraction and synthesis, and quality assessment was followed.

### Search strategy for identification and selection of publications

The aim of the search strategy was to provide a comprehensive list of published literature reviews assessing decision making capacity in patients with mental disorders. Four electronic databases (the Cumulative Index to Nursing and Allied Health Literature [CINAHL], PsycInfo, PubMed and Scopus) were searched up to 31 January 2020. The search strategy is described in Additional file 1. Free text searches and medical subject headings were combined to identify literature reviews published in English, summarising studies conducted in any clinical setting and with a quantitative synthesis of results. Selection of publications was carried out as per inclusion and exclusion criteria (Table 1). Lists of references in the key papers retrieved were further checked to identify other relevant articles.

**Table 1:** Selection criteria

Inclusion criteria:	Exclusion criteria
Topics: decision-making capacity regarding medications/pharmacological treatment/healthcare Population: mental health/illness, psychiatric disorders/psychosis/schizophrenia/bipolar disorder Type of study: any review of the literature with a quantitative synthesis of results Language of publication: English. Setting: any (inpatient, outpatient, forensic)	Animals, in-vitro, or other types of pre-clinical study Studies on dementia, depression, Down syndrome, attention deficit- hyperactivity disorders, autism spectrum disorders, learning-, sleep-, eating-hoarding-, gambling- personality- or dissociative disorders Studies of decision-making in presence of tumours of the central nervous system; cognition deficits occurring in the context of progressive chronic diseases (e.g., multiple sclerosis, cardiovascular, respiratory, infection diseases) Studies solely of the healthcare decision-making of professionals and carers of persons with mental disorders Studies on health- and social-care services provision planning Studies on factors determining healthcare decision-making capacity Studies on shared decision making (decisional capacity not assessed) Studies on interventions devoted to improving decision-making capacity in mental disorder patients (decisional capacity not assessed) Studies on capacity to consent to research Intellectual, developmental and learning disability studies New-borns, infants, children or adolescent studies Tool studies Clinical practice guidelines Conceptual model studies

Potentially relevant abstracts were assessed by two expert reviewers to identify all papers suitable for inclusion. Full text copies were requested. Reviews which were identified after mutual agreement were included and data were extracted. A third reviewer was involved in the process to resolve any disagreements on the selection of publications.

### Data extraction and quality assessment

Data extraction was carried out by one researcher. A data extraction form that covered citation, country, population, interventions, comparators, outcomes, settings, review type, aims, literature review size, strengths and limitations and key findings of the review as stated by authors was used to extract data (Tables 2 and 3). The AMSTAR II (A MeaSurement Tool to Assess systematic Reviews) [22] assessment tool was used to assess the quality of reviews.

**Table 2:** Summary of the PICOS concepts in the reviewed publications

Study	Country	PICOS						
		Population	N	Intervention	Comparator	N	Outcomes	Setting
Eiring Ø et al. 2015, [11]	Norway	Schizophrenia, depression, bipolar disorder, attention deficit hyperactive disorder	1785	Stated preferences by means of willingness to pay, conjoint analysis, discrete choice experiment	None	–	Relative value placed on treatment outcomes.	Inpatients, outpatients
Hostiuc S et al. 2018, [12]	Romania	Schizophrenia	684	MacArthur Competence Assessment Tool -Clinical Research	Non-mental illness patients (control)	548	Differences in MacArthur Competence Assessment Tool measurements and consent to research participation	Inpatients, outpatients
Jeste DV et al. 2006, [13]	United States	Schizophrenia	Range: 6–80	MacArthur Competence Assessment Tool -Treatment -Clinical Research	Non-psychiatric comparison subjects	Range: 15–82	Differences on MacArthur Competence Assessment Tool	Inpatients and outpatients
Larkin A et al. 2017, [14]	United Kingdom	Psychosis (non-affective psychotic disorder)	1823	Brief Psychiatric Rating Scale. Positive and negative syndrome scale score	None	–	Factors that help or hinder treatment decision-making capacity. Assessment of direction, magnitude, significance and reliability of reported associations	Inpatients and outpatients
Lepping P et al. 2015, [15]	United Kingdom	Psychiatric disorder patients	2483	Any validated tool to assess capacity for making decisions	Non-psychiatric (medical) patients	1710	Incapacity to consent to treatment or admission	Inpatients, outpatients, forensic
Mukherjee D et al. 2014, [16]	United States	Mental illness patients	1813	Iowa Gambling Task	Healthy individuals Individuals with frontal lesions	3165	First meta-analysis: effectsizes for comparisons between healthy individuals and those with mental illness Second meta-analysis: raw scores from the IGT across different mental illnesses	Outpatients
Okai D et al. 2007, [17]	United Kingdom	Psychiatric disorder patients	851	Vignettes, 15-item questionnaire, MacArthur Competence Assessment Tool- Treatment, semi-structured interview	None	–	Capacity to consent to treatment	Inpatients and outpatients
Ruissen AM et al. 2011, [18]	Netherlands	Psychotic and non-psychotic disorder patients	735	MacArthur Competence Assessment Tool	None	–	Competence and treatment decision and insight	Inpatients and outpatients
Spencer BWJ et al. 2017, [19]	United Kingdom	Schizophrenia and non-affective disorders	Not reported	MacArthur Competence Assessment Tool -Treatment -Clinical Research	None	–	Decision making capacity for treatment and research	Inpatient and outpatients
Wang SB et al. 2017, [20]	Multicountry	Schizophrenia or schizoaffective disorder	422	MacArthur Competence Assessment Tool -Clinical Research -Treatment	Healthy controls	304	Decision making capacity	Outpatients
Woodrow A et al. 2018, [21]	United Kingdom	Psychosis	2276	Iowa and Cambridge Gambling Tasks	Healthy controls	1988	Between-group comparison of performance on IGC instruments	Outpatients

**Table 3:** Summary of significant characteristics and findings of reviewed publications based on literature review domains

Reference	Literature review domains						
	Review type	Aims/objectives	Literature review size, n	Key findings/conclusions (effect sizes/associations)	AMSTAR II score^a^	Strengths	Limitations
Eiring Ø et al. 2015, [11]	Systematic review	To investigate patients’ preferences for outcomes associated with psychoactive medications.	16	Side effects and symptom outcomes outnumbered functioning and process outcomes. Severe disease and hospitalisation were reported to be least desirable. Patients with schizophrenia tended to value disease states as higher and side effects as lower, compared to other stakeholder groups. In depression, the ability to cope with activities was found to be more important than a depressed mood. Patient preferences could not be consistently predicted from demographic or disease variables.	High	First systematic review on patients’ stated preferences for outcomes of psychopharmacological treatments across methods and disorders.	Preferences for outcomes were elicited from varying and often small numbers of participants, with heterogeneous disorders. Owing to the heterogeneity of methods and outcomes, quantitative summaries of the relative strengths of preferences could not be performed
Hostiuc S et al. 2018, [12]	Systematic review	To evaluate the degree of impairment in each dimension of decision-making capacity in schizophrenia patients compared to non-mentally-ill controls, as quantified by the MacArthur Competence Assessment Tool for Clinical Research instrument.	13	Effect size: differences in means, schizophrenia vs non-mental illness patients: Understanding: −4.43 (−5.76; −3.1, *p* < 0.001) Appreciation: −1.17 (−1.49, − 0.84, *p* < 0.001) Reasoning: − 1.29 (− 1.79, − 0.79, *p* < 0.001) Expressing a choice: − 0.05 (− 0.9, − 0.01, *p* = 0.022) The odds for a decreased understanding in schizophrenia patients were about five times higher than in control groups (OR = 0.18, 95% CI: 0.12; 0.29, *p* < 0.001).	High	Despite the small number of studies, the results reached statistical significance in most scales, suggesting that enhanced informed consent forms profoundly improved decision-making capacity	Small number of reviewed studies.
Jeste DV et al. 2006, [13]	Narrative review	To evaluate the magnitude of the difference between schizophrenia and non- psychiatric comparison subjects reported as well as the influence of sample characteristics on the effect sizes observed.	12	Schizophrenia vs non-psychiatric comparison subjects Effect size: Understanding subscale (mean d = 0.88, SD = 0.40, 7 studies), Appreciation subscale (mean d = 0.93, SD = 0.34, 4 studies), Reasoning (mean d = 0.65, SD =0.34, 7 studies), Expression of choice (mean d = 0.29, SD = 0.24, 4 studies). Psychopathology (mean d = 2.06, SD = 1.03, 4 studies). Cognition (mean d = 1.01, SD = 0.61, 6 studies). Effect size comparing different MacArthur scales amongst inpatients with schizophrenia to non- psychiatric comparison subjects: range 0.45–1.54; median = 1.17) Effect size comparing different MacArthur scales amongst clinically stable outpatients with schizophrenia to non-psychiatric comparison subjects: range 0.0–0.84, median = 0.53	High	The review of studies comparing structured measures of decision-making capacity between persons with schizophrenia and non-psychotic medical individuals revealed considerable variability in study design in terms of sample sizes, setting, age range of the participants, measures used to assess capacity, and the nature of the proposed study or intervention.	Decisional capacity is a context specific construct, and it is difficult to compare the absolute magnitude of decisional capacity scores across studies.
Larkin A et al. 2017, [14]	Systematic review	To determine the direction, magnitude and reliability of the relationship between capacity in psychosis and a range of clinical, demographic and treatment-related factors	23	Association between total psychotic symptoms and capacity to understand information relevant to treatment decisions: *r* = − 0.45 (95% CI − 0.55 to − 0.34; =60%) Correlation between overall symptoms and ability to appreciate information for treatment decision *r* = − 0.23 (95% CI − 0.14 to − 0.32; =0%) Correlation between total symptoms and ability to reason in relation to treatment decision making *r* = − 0.31 (95% CI − 0.48 to − 0.12;=80%)	High	Only studies that included a reliable and valid assessment of capacity in adults diagnosed with a non-affective psychotic disorder and provided data on the association between capacity and at least one other clinical or demographic variable were included	Capacity was treated as a continuous variable in meta-analyses, even though in legal and clinical practice binary decisions must be made. The correlational nature of much of the data in the meta-analysis limits a definitive assessment of causality.
Lepping P et al. 2015, [15]	Systematic review	To estimate the prevalence of incapacity to consent to treatment or admission in different medical and psychiatric settings, and compare the two	58 (35, psychiatric settings. 23, medical, non-psychiatric settings)	Inverse variation weighted prevalence for decision-making capacity for all the studies included was 41% (95% CI 35.6–46.2%). Heterogeneity was significant (Cochran Q 601; (df) 69; *p* < 0.001). Psychiatric settings: the inverse variance weighted proportion of patients with incapacity was 45% (95% CI 39–51%). Heterogeneity was significant (Cochran Q 300; df 42; *p* < 0.001). Medical settings: the inverse variance weighted proportion of patients with incapacity was 34% (95% CI 25–44%). Heterogeneity was significant (Cochran Q 267; df 26; *p* < 0.001), with inconsistency I2 at 90% (95% CI 87–93%). Variation between studies due to heterogeneity was 90%. Psychiatric and medical settings did not differ significantly from each other in terms of the proportion of incapacity (Cochran Q 0.66; df 1; *p* = 0.44)	High	Only studies with valid measurement tools were included	High level of heterogeneity between studies. Cut-off points for various tools are still being investigated. Studies were not weighted according to their quality
Mukherjee D et al. 2014, [16]	Systematic review	To assess value-based decision making in individuals diagnosed with mental illness.	63, first meta-analysis (healthy populations - and individuals with frontal lesions - and populations with mental illness) 40, second meta-analysis (as a function of type of mental illness)	Individual study effect sizes ranged from 0 to − 1.55 Mean effect size was −0.58 (95% CI = − 0.68 to − 0.48, *p* < .001). Population with lesions performed significantly worse than the population with mental illness. Q (1) = 6.57, *p* = .01, d = 0.52. Population with mental illness, mean performances in individual studies ranged from − 6.72 to 10.20, average 0.45 (SE = 0.88)	High	The current meta-analyses provide a broad screen for possible impairments in value-based decision making by assessing Iowa Gambling Task performance across mental illnesses.	Limitations inherent to the Iowa Gambling Task (e.g. not specific for any single decision process)
Okai D et al. 2007, [17]	Systematic review	To describe the clinical epidemiology of mental incapacity in patients with psychiatric disorders, including interrater reliability of assessments, frequency in the psychiatric population and associations of mental incapacity	37	Psychiatric in-patients lacking capacity reached 29% (median) (IQR 22–44). 67% had mental capacity to make decisions regarding admission to a psychiatric unit	High	Studies report that most psychiatric in-patients can make key treatment decisions. Studies are consistent in showing the reliability of mental capacity assessments; these measurements are correlated with indicators of clinical severity but not with demographic differences.	Heterogeneity of the patient groups, wide range of capacity assessment tools used, different legal standards for capacity assessment, differences in treatment choices presented to participants. Frequency of capacity in some of the primary research was not the main aim of the study and was reported as an incidental finding. Studies were often small, and many used convenience samples.
Ruissen AM et al. 2011, [18]	Systematic review	To review the scientific literature on the relationship between competence and insight in patients with psychiatric disorders, how competence and insight are connected in these patients, and whether there are differences in competence and insight among patients with different disorders	7	Psychotic patients with poor insight are very likely to be incompetent Psychotic patients with adequate insight are generally competent. In non-psychotic patients, competence and insight do not completely overlap. Most incompetent patients in this group have poor insight, but a substantial number of non-psychotic patients with adequate insight were incompetent. Non-psychotic patients with adequate insight can be incompetent.	High	Provides evidence on the correlation between competence and insight in a wide range of mental disorders	Only English-language articles were included. One article did not report a relevant and significant correlation between insight and competence, producing less convincing results.
Spencer BWJ et al. 2017, [19]	Qualitative systematic review	To examine the presence or absence of decision-making capacity in schizophrenia and the associated socio-demographic/psycho-pathological factors.	40	Decision-making capacity was present in 48% of people (range: 26–67%).	High	Provides robust evidence that a significant proportion of people with schizophrenia, even on inpatient wards, have decision-making capacity; that decision-making capacity is associated with clinically relevant variables, such as insight and neurocognitive performance,that it is not related to socio-demographic factors.	Use of dimensional measures of decision-making capacity in isolation in all studies
Wang SB et al. 2018 [20]	Systematic Review	To examine the decisional capacity measured by the MacArthur Competence Assessment Tools in schizophrenia.	7	Decision-making capacity in schizophrenia patients compared to the healthy control Understanding (SMD = −0.81, 95% CI: − 1.06 to − 0.56, *p* < 0.001), Reasoning (SMD = − 0.57, 95% CI: − 0.80 to − 0.34, *p* < 0.001), Appreciation (SMD = − 0.87, 95% CI: − 1.20 to − 0.53, *p* < 0.001) Expression a choice (SMD = − 0.24, 95% CI: − 0.43 to − 0.05, *p* = 0.01).	High	Provides consistent evidence that schizophrenia patients appear to have impaired decision-making competence in medical research and treatments.	The sample size of the included studies was relatively small. Different MacCAT versions were used across studies. Patients with severe medical conditions interfering with their ability to complete the assessments were excluded. Frequent failure to blindly assess decision making capacity, which may have biased the results
Woodrow A et al. 2018, [21]	Systematic review	To identify factors that may help or hinder decision-making ability in people with psychosis measured with Iowa or Cambridge Gambling Tasks	50	People with psychosis: -had moderately impaired decision-making ability compared with non-clinical individuals: g = − 0.57, 95% CI − 0.66 to − 0.48; I2 45% (moderate quality) -were significantly more likely than healthy individuals to value rewards over losses: k = 6, *N* = 516, g = 0.38, 95% CI 0.05–0.70, I2 64%. Within the psychosis groups, decision-making performance had: -a small-moderate inverse association with negative symptoms: k = 13, *N* = 648, *r =* − 0.17, 95% CI − 0.26 to − 0.07, I2 32% (moderate quality), -a small association with general symptoms: k = 5, *N* = 169, *r* = − 0.13, 95% -0.25, − 0.00, I2 = 0% (low quality) -no association with positive symptoms: k = 10, *N* = 512, *r* = − 0.01, 95% CI − 0.11 to 0.08 (moderate quality) -no association between overall psychotic symptoms: k = 6, *r* = − 0.10, 95% CI − 0.21 to 0.02, I2 = 0% (very low quality). -no association with current antipsychotic doses: *N* = 171, *r* = − 0.02, 95% CI − 0.17 to 0.13, I2 = 0% (low quality)	High	Provides evidence that people with non-affective psychosis appear to make less effective decisions than healthy individuals when this is assessed using the Iowa or Cambridge Gambling Tasks. The moderate difficulties they have are comparable with those observed in other clinical groups	Small sample sizes Limitations inherent to the Iowa or Cambridge Gambling Tasks to assess decision-making capacity in mental health patients.

^a^AMSTAR II Score, interpretation: High- Zero or one non-critical weakness: The systematic review provides an accurate and comprehensive summary of the results of the available studies that address the question of interest; Moderate- More than one non-critical weakness. The systematic review has more than one weakness, but no critical flaws. It may provide an accurate summary of the results of the available studies that were included in the review.; Low - One critical flaw with or without non-critical weaknesses: The review has a critical flaw and may not provide an accurate and comprehensive summary of the available studies that address the question of interest; Critically low - More than one critical flaw with or without non-critical weaknesses: The review has more than one critical flaw and should not be relied on to provide an accurate and comprehensive summary of the available studies

*CI* confidence interval, *IQR* InterQuartile Range, *OR* Odds Ratios, *NNT* Number Needed to Treat, *SD* Standard Deviation, *SE* Standard Error, *SMD* Standardized Mean Difference

## Results

**Fig. 1:**
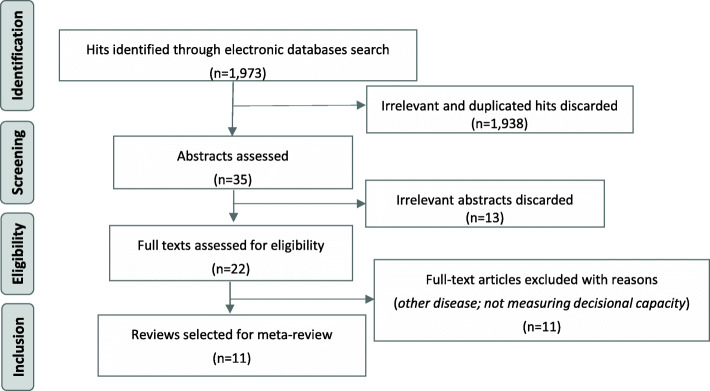
PRISMA diagram

A total of 1973 hits were initially identified; 1938 were either duplicated or deemed not relevant for the review based on the assessment of titles and abstracts; 22 full text publications were initially considered valid and retrieved for closer examination; 11 were excluded because they referred to diseases excluded from the review or did not assess decision making capacity. Data was finally extracted from 11 publications (Fig. 1).

The number of studies included in each review apprised varied between 7 [18, 20] and 63 [16], and the number of patients with a mental disorder ranged from 6 [13] to 2483 [15]. Schizophrenia or schizoaffective disorders [12, 13, 19, 20] and psychosis [14, 18, 21] were the most frequently explored mental illnesses. The general healthy population or patients with a non-mental disorder were the usual study comparators. Therefore, decisional capacity variance among mental illnesses of different nature has been little explored (Tables 2 and 3).

### Prevalence of decision-making capacity

Information on the prevalence of capacity for making healthcare decisions among psychiatry patients can be derived from two systematic reviews. One systematic review of 37 empirical, quantitative studies of mental capacity in a mixed population of psychiatric patients reported that up to 67% of participants had the capacity to decide whether to be admitted to a psychiatric unit while a median of 71% had capacity for making treatment decisions (a median of 29%, interquartile range (IQR) 22–44, lacked capacity) [17]. Another systematic review (40 articles) found that 26% (95% confidence interval (CI): 18 to 36) to 67% (95% CI: 35 to 88) of people with schizophrenia or other non-affective disorders were able to make medical decisions related or unrelated to the management of their condition [(median: 48% (95% CI: 29 to 66)] [19]. In both reviews, up to three quarters of severe mental disorder patients, including individuals with schizophrenia would have capacity to make medical decisions in the context of their illness, in particular specific decisions related with their treatments [17, 19]. Both reviews are also coincident in that heterogeneity between studies was high, with considerable variation in study design and measurements [17, 19].

### Decisional capacity in different clinical settings and patient groups

Four reviews assessed patients in diverse settings and explored the degree of impairment in each dimension of decision-making capacity. Lepping et al. [15] reported that 55% of patients in psychiatric and 66% of patients in non-psychiatric settings had the capacity to make medical decisions. Appreciation of the problem and necessity for treatment were more frequently compromised in psychiatric patients, while non-psychiatric patients struggled primarily with reasoning. The authors found a significant variation between studies due to heterogeneity in designs and methods that reached 86% in psychiatric settings and 90% in non-psychiatric settings. Jeste et al. [13] reported a 48 to 79% overlap between people with schizophrenia and non-psychiatric patients on the MacArthur subscales, which indicated that most patients with schizophrenia had comparably adequate decision-making capacity [13]. Psychotic inpatients had several characteristics which temporarily limited their capacity and distinguished them from outpatients. Greater severity of positive and negative symptoms, experiencing a stressful life event (e.g., hospitalisation), and often receiving higher doses of medication adversely impacted cognition among psychiatric inpatients [15]. Community-dwelling or clinically stable outpatients were much closer to non-psychiatric subjects in terms of the capacity for decision-making. The authors concluded that similar proportions of non-psychiatric and psychiatric outpatients either had or lacked capacity to consent to treatment or to hospital admission, and that impairment in the capacity to make decisions was not a distinguishing feature of schizophrenia patients [13, 15].

Another meta-analysis of ten studies showed that compared to healthy controls, patients with schizophrenia or schizoaffective disorder were significantly more likely to have impaired decision-making capacity in terms of understanding, reasoning, appreciation and expression of a choice in clinical research and treatment, as measured by the MacArthur Competence Assessment Tool (MacCAT) instruments [20]. The standardised mean differences were more significant in older than in younger age subgroups, suggesting that, compared to their healthy counterparts, the impairment of decision-making capacity could be more obvious in older patients than in younger patients. In some of the studies included in this meta-analysis, decisional capacity improved in patients with schizophrenia following intensive educational interventions.

Another systematic review explored the degree of impairment in each dimension of decision-making capacity in schizophrenia patients compared to non-psychiatric controls, as assessed by the MacCAT [12]. The odds for a decreased understanding and a decreased appreciation were some five times higher in individuals with schizophrenia than in non-mentally ill controls, those for decreased reasoning almost four times higher, and those for a decreased aptitude to express a choice was over six times higher. The use of an enhanced informed consent form contributed to significant improvements in decision-making capacity compared to the use of standard forms. The authors concluded that even if patients with schizophrenia have a significantly decreased decision-making capacity, they should be considered to be as competent as non-mentally ill controls unless very severe changes were identifiable during the clinical examination [12].

In these four systematic reviews, the decisional capacity of patients with a psychiatric disease was compared with that of patients with a non-psychiatric clinical condition or with that of healthy individuals. All reviews are concurrent in the fact that impairments in decisional capacity can be found in both psychiatric and non-psychiatric patients, and therefore the diagnosis of a psychiatric condition should not be the upfront reason of incapacity. Despite most research being conducted in the hospital setting, those fewer addressing decisional capacity in psychiatric outpatients showed that their capacity to make medical decisions can be much alike to that of the non-psychiatric individuals. Likewise, studies coincidently acknowledge that decisional impairments amongst psychiatric inpatients are temporal and responsive to information-enhancing interventions.

### Determining factors of decisional capacity in psychosis patients

In a systematic review and meta-analysis of factors that help or hinder treatment decision-making capacity in psychosis (23 studies, *n* = 1823) a moderate to large negative association between total psychotic symptom severity and the capacity of participants to understand information relevant to treatment decisions was found [14]. Poor insight was also associated with patients’ poor capacity to make treatment related decisions. Verbal cognitive function, metacognitive ability and years spent in education were positively associated with the ability of psychiatric individuals to understand information relating to treatment decision making. Decision-making capacity responded favourably to interventions, such as the simplification of the information, shared decision-making, and metacognitive training [14].

Likewise, Ruissen et al. [18] reported the findings of seven articles that assessed the relationship between competence to decide and insight of psychiatric inpatients and outpatients and of psychotic and non-psychotic patients. A large overlap between insight and competence to decide was reported among psychotic (schizophrenia, schizoaffective disorder, and psychotic episodes) and bipolar disorder (comprising both manic and depressive episodes) patients implying that a strong correlation existed between insight and capacity for making decisions, including decisions related to medical treatments and hospital admission. Psychotic patients with adequate insight were generally competent in making medical decisions.

Both reviews report findings on the capacity of psychotic patients to make treatment and other disease-related decisions. They are coincident in the relevance of insight as a determining factor of psychiatric patients’ decisional capacity. As expected, the burden and severity of psychotic symptoms can seriously compromise patients’ ability to make decisions.

### Capacity of people with mental illness to make risk-reward decisions and to choose treatments

The capacity of mental illness patients for making value-based decisions was explored in literature reviews of studies based on gambling tasks and on preferences for medication-associated outcomes methods. A systematic review and meta-analysis explored the factors which may help or hinder the ability to make risk-reward decision making in a pooled sample of 4264 individuals with psychosis, based on their performance on the Iowa Gambling Tasks (IGT) and the Cambridge Gambling Tasks (CGT) [21, 23, 24]. Compared with healthy individuals, people with psychosis had moderately impaired risk-reward decision-making ability (g = − 0.57, 95% CI − 0.66 to − 0.48; I^2^ 45%; moderate quality) [21]. They were also more likely to value rewards over losses (k = 6, *N* = 516, g = 0.38, 95% CI: 0.05 to 0.70, I^2^ 64%), and to base decisions on recent rather than past outcomes (k = 6, *N* = 516, g = 0.30, 95% CI: − 0.04 to 0.65, I^2^ 68%). Analysis of the positive or negative influence of the type and dose of antipsychotics on decision-making capacity was inconclusive. The authors suggested that, although people with non-affective psychosis may make less effective decisions than healthy individuals in the IGT and CGT, their difficulties were moderate and comparable with those observed in other clinical groups.

Mukherjee and Kable [16] calculated that around 27% of patients with various mental disorders were similar to healthy individuals when deciding about losses and rewards on the IGT. Furthermore, individuals with mental illnesses had fewer deficits than individuals with frontal lobe lesions, for instance. The assessment of the severity of impairment across types of mental illnesses did not demonstrate any significant differences according to specific psychiatric diagnosis.

Eiring et al. [11] investigated the relative value adults with a mental illness place on treatment outcomes, including the attributes of particular medications or medication classes and the consequences and health states associated with their use. It reported that patients were able to provide valid preference measures with the different methods applied, generally understood the tasks, and gave sufficiently consistent answers. Among patients with schizophrenia, positive, acute or psychotic symptoms appeared consistently among the least desirable outcomes. Negative symptoms, such as reduced capacity for emotion, were found more desirable or less important than positive symptoms. Independence received high ratings and inpatient status low ratings. Overall, patients with schizophrenia tended to value disease states higher and side effects lower than other groups and perceived side effects more negatively than their therapists. Patients with bipolar disorder gave low values to mania and severe depression and reported weight gain to be important.

These reviews provide consistent evidence on the fact that patients with a serious mental disease, such as schizophrenia or bipolar disorder can make risk-reward decisions in the context of their illness and treatments. Furthermore, the studies summarised in the reviews show that these patients may achieve a level of ability for making value-based decisions equal to non-psychiatric patients. They can reliably decide about the important treatment outcomes and their most desirable treatment attributes.

### Quality assessment

Reviews presented well-framed research questions based on the evidence based PICOS model [25] (Table 2) and were high quality according to the AMSTAR II assessment tool (Table 3) [22]. AMSTAR II was developed to evaluate systematic reviews of randomised trials or non-randomised studies of healthcare interventions, or both. Publications included in this review complied satisfactorily the AMSTAR II critical domains. No critical weaknesses were identified in the assessment. Therefore, the reviews provided an accurate and comprehensive summary of the results of studies of decision-making capacity in mental disorder patients.

## Discussion

This meta-review review brings together a set of high-quality reviews on the capacity of individuals with a severe mental illness to make decisions about their healthcare. It presents a thorough synthesis of current systematic review literature concerning the decision-making capacity of patients with mental disorders, including psychotic, schizophrenia and bipolar disorder individuals. It provides a picture of the state of the field in the complex task of assessing patients’ decisional capacity in psychiatry. It comprehensively summarizes a body of evidence supporting the idea that the decision-making capacity of psychiatric patients with serious mental illness is preserved in most circumstances and challenges the understanding that people with severe mental illnesses are unable to make their own choices [8].

Authors across studies are coincident in emphasising that most patients with a severe mental disorder are able to make rational decisions about their medical care and to participate in decision-making regarding treatments despite temporal impairments. Thus, most often the degree of impairment that may be inherent to the mental disorder does not constitute incapacity to make decisions. The findings also reveal that patients with psychotic disorders or other severe mental illnesses can make complex risk-reward decisions in usual clinical practice. Small deviations from optimal performance may arise due to deficits in the ability to fully represent the value of different choices and response options, a finding that aligns with results from experimental research in patients with schizophrenia [26].

Most of the reviews addressed the capacity to make decisions in people with severe mental disorders either already hospitalised or requiring hospital admission. This means that most studies included patients with more severe symptoms less responsive to usual therapies [27]. Even in these more ill psychiatric populations, between 60 and 70% had capacity to make some treatment decisions [1, 17, 19]. Hospitalised patients usually have greater care needs, even when their psychiatric symptoms are controlled, exhibit significantly more severe negative, positive, and manic symptoms, and have lower global functioning than outpatients [28]. Therefore, despite the scarcity of studies measuring decisional capacity in routine ambulatory practice in psychiatry, it can be expected a high level of health-related decision-making capacity in patients in everyday life in the community. Rigorous studies investigating this question as a primary outcome would be much welcome.

This meta- review also shows that people with schizophrenia have the capacity to make other difficult decisions related, for instance, to giving consent for hospital admission or to the type of treatment they prefer to receive. Likewise, other studies have reported that patients with schizophrenia or bipolar disorder are able to describe prodromal symptoms of relapse and to suggest a treatment and the need for hospitalisation in advance; that they can request or refuse medications and state their preferences for pre-emergency interventions, non-hospital alternatives and non-medical personal care [29–31]. In such circumstances, shared decision making and advancing crisis management plans may help reduce insecurity and improve healthcare outcomes for the patient with a mental illness. Studies have found that being involved in decision-making, whenever decisional capacity exists, renders more positive treatment results, better medication adherence, higher self-efficacy and autonomy, and lower decisional uncertainty among patients with mental disorders [32, 33].

Patients’ awareness of decisional capacity and having the opportunity for sharing decisions on future care (crisis planning) for psychosis reduces the use of compulsory inpatient treatment by approximately 40% over 15 to 18 months [33]. In this context, advance directives are fundamental to ensure the timely provision of medical treatments, thus minimising decisional impairments in the acute stages of psychosis [34]. Psychosocial interventions are also important to address the complex health needs of people with serious mental illnesses. Combined with anti-psychotics, psychosocial interventions highly contribute to reduce the severity of symptoms, to benefit functioning, to encourage decision making and to decrease hospital readmissions [35].

Nevertheless, clinicians play the crucial role of judging the capacity of patients with severe mental disorder to decide about their treatments and healthcare, and tools exist to guide their assessment [36]. The final decision depends entirely on clinical judgement, based on the practitioner’s knowledge of the patient and of the course of the disease.

Beyond acute episodes, the findings also support the notion that continued training and learning, simplification and enhancement of the information improve the capacity of patients with severe mental disorders for decision-making both in hospital and in everyday life [37]. The results of various studies demonstrate that brief interventions aimed at recovering capacity for understanding can help schizophrenia patients to perform very much like healthy people in the four dimensions of decisional capacity (understanding, appreciation, reasoning and expression of a choice) [38]. Regular information reinforcement, strengthening neurocognitive functioning and training are important to maintain long-term levels of competence and to maximise decision making capacities of patients [39, 40].

In sum, people with severe mental illness can benefit greatly from anticipation, prevention, gradual learning, enhanced information and enriched shared decision-making in order to strengthen their autonomous decision-making capacity, to increase their autonomy and to ultimately contribute to reducing the stigma of mental illness. Being able to make decisions in anticipation of, for instance, agitation or other acute symptoms should help patients to gain a sense of control over their own lives, and to enhance their health-related quality of life [41]. This review contributes to the growing body of evidence suggesting that the best medical practice should help severe mentally ill patients to grow into voluntary healthcare, safe users of medications.

Small sample size, heterogeneity, language and selection bias of participants were among the limitations frequently reported by the authors of the studies reviewed. However, since the publications included in the meta- review were systematic reviews and meta-analyses, the risk of bias was minimised. Nonetheless, it was limited to publications appeared in English. Although the search was comprehensive, papers in many other languages including French, Germany. Italian or Spanish may not have been identified. Several frequent mental illnesses were excluded for reasons of study feasibility. Important mental health conditions, such as dementia, depression and other disorders have not been addressed which may have limited the scope of the meta-review.

## Conclusions

This meta-review of review articles provides a comprehensive synthesis of the current state of knowledge on the capacity of patients with mental illnesses to make decisions about their healthcare and medical treatments. It provides clinicians and other healthcare practitioners a summary of the evidence on the topic, contrasting key findings. It shows that whilst impairments in decision-making capacity may exist, most patients with a severe mental disorder are able to make adequate decisions about the care of their health. It denotes that best practice strategies should help mentally ill patients to exercise their decisional capacity to develop into autonomous and reliable users of medications. Keeping a sense of control over their illness and life may help them to improve the outcomes of their treatments and their health-related quality of life.

## Supplementary information

**Additional file 1.** Meta- review search strategy.

## Data Availability

The datasets used and/or analysed during the current study are available from the corresponding author on reasonable request.

## Abbreviations

Abreviation: Description
AMSTAR: A MeaSurement Tool to Assess systematic Reviews
CGT: Cambridge Gambling Tasks
CI: Confidence Interval
CINAHL: Cumulative Index to Nursing and Allied Health Literature
IGT: Iowa Gambling Tasks
IQR: InterQuartile Range
MacCAT: MacArthur Competence Assessment Tool
MacCAT-CR: MacArthur Competence Assessment Tool for Clinical Research
MacCAT-T: MacArthur Competence Assessment Tool for Treatment
NNT: Number Needed to Treat
OR: Odds Ratios
PRISMA: Preferred Reporting Items for Systematic Review and Meta-Analysis
SD: Standard Deviation
SE: Standard Error
SMD: Standardized Mean Difference
