# Supplementary material for: A meta-review of literature reviews assessing the capacity of patients with severe mental disorders to make decisions about their healthcare
Source: BMC Psychiatry. 2020 Jun 30;20:339. doi: 10.1186/s12888-020-02756-0 (PMC7324958; doi:10.1186/s12888-020-02756-0)
Supplement: Supplementary file 1 [file 12888_2020_2756_MOESM1_ESM.odt]

Appendix 1

Meta- review search strategy

|  | Databases | CINAHL, hits (n) | PubMed, hits (n) | PsycInfo, hits (n) | Scopus, hits (n) |
| --- | --- | --- | --- | --- | --- |
| # | Search terms, strategy |  |  |  |  |
|  | Limit #9 #13 #19 #20 #22 #23 to English language, Review [any], Journal article, Humans |  |  |  |  |
| 1 | mental illness |  |  |  |  |
| 2 | mental disorder |  |  |  |  |
| 3 | psychiatric illness |  |  |  |  |
| 4 | psychiatric disorder |  |  |  |  |
| 5 | decision making capacity |  |  |  |  |
| 6 | healthcare |  |  |  |  |
| 7 | health care |  |  |  |  |
| 8 | medical care |  |  |  |  |
| 9 | #1 OR #2 OR #3 OR #4 AND #5 AND #6 OR #7 OR #8 | 10 | 373 | 11 | 86 |
| 10 | treatment |  |  |  |  |
| 11 | intervention |  |  |  |  |
| 12 | therapy |  |  |  |  |
| 13 | #1 OR #2 OR #3 OR #4 AND #5 AND #8 OR #10 OR #11 OR #12 | 26 | 732 | 21 | 173 |
| 14 | schizophrenia |  |  |  |  |
| 15 | psychosis |  |  |  |  |
| 16 | psychoses |  |  |  |  |
| 17 | psychotic disorder |  |  |  |  |
| 18 | schizophrenic disorder |  |  |  |  |
| 19 | #14 OR #15 OR #16 OR #17 OR #18 AND #5 AND #10 OR #11 OR #12 | 0 | 82 | 8 | 150 |
| 20 | #14 OR #15 OR #16 OR #17 OR #18 AND #5 AND #6 OR #7 OR #8 | 0 | 66 | 0 | 68 |
| 21 | bipolar disorder |  |  |  |  |
| 22 | #21 AND #5 AND #10 OR #11 OR #12 | 0 | 55 | 0 | 44 |
| 23 | #21 AND #5 AND #6 OR #7 OR #8 | 0 | 44 | 0 | 24 |
|  |  |  |  |  |  |
| Total hits per database (all search strategies) | 36 | 1,352 | 40 | 545 |
| Total hits | 1,973 |
